# Targeted Metabolomics Identifies Reliable and Stable Metabolites in Human Serum and Plasma Samples

**DOI:** 10.1371/journal.pone.0089728

**Published:** 2014-02-24

**Authors:** Michaela Breier, Simone Wahl, Cornelia Prehn, Marina Fugmann, Uta Ferrari, Michaela Weise, Friederike Banning, Jochen Seissler, Harald Grallert, Jerzy Adamski, Andreas Lechner

**Affiliations:** 1 Research Unit of Molecular Epidemiology, Helmholtz Zentrum Muenchen, German Research Center for Environmental Health, Neuherberg, Germany; 2 Institute of Epidemiology II, Helmholtz Zentrum Muenchen, German Research Center for Environmental Health, Neuherberg, Germany; 3 German Center for Diabetes Research, Neuherberg, Germany; 4 Clinical Cooperation Group Subclassification of Type 2 Diabetes, Ludwig-Maximilians-Universität Muenchen and Helmholtz Zentrum Muenchen, Munich, Germany; 5 Institute of Experimental Genetics, Genome Analysis Center, Helmholtz Zentrum Muenchen, German Research Center for Environmental Health, Neuherberg, Germany; 6 Medizinische Klinik und Poliklinik IV, Diabetes Research Group Klinikum der Universität München, Munich, Germany; 7 Chair for Experimental Genetics, Technical University of Munich, Freising-Weihenstephan, Germany; Aligarh Muslim University, India

## Abstract

**Background:**

Information regarding the variability of metabolite levels over time in an individual is required to estimate the reproducibility of metabolite measurements. In intervention studies, it is critical to appropriately judge changes that are elicited by any kind of intervention. The pre-analytic phase (collection, transport and sample processing) is a particularly important component of data quality in multi-center studies.

**Methods:**

Reliability of metabolites (within-and between-person variance, intraclass correlation coefficient) and stability (shipment simulation at different temperatures, use of gel-barrier collection tubes, freeze-thaw cycles) were analyzed in fasting serum and plasma samples of 22 healthy human subjects using a targeted LC-MS approach.

**Results:**

Reliability of metabolite measurements was higher in serum compared to plasma samples and was good in most saturated short-and medium-chain acylcarnitines, amino acids, biogenic amines, glycerophospholipids, sphingolipids and hexose. The majority of metabolites were stable for 24 h on cool packs and at room temperature in non-centrifuged tubes. Plasma and serum metabolite stability showed good coherence. Serum metabolite concentrations were mostly unaffected by tube type and one or two freeze-thaw cycles.

**Conclusion:**

A single time point measurement is assumed to be sufficient for a targeted metabolomics analysis of most metabolites. For shipment, samples should ideally be separated and frozen immediately after collection, as some amino acids and biogenic amines become unstable within 3 h on cool packs. Serum gel-barrier tubes can be used safely for this process as they have no effect on concentration in most metabolites. Shipment of non-centrifuged samples on cool packs is a cost-efficient alternative for most metabolites.

## Introduction

The inclusion of the serum or plasma metabolome analysis in clinical trials is an appealing approach for several reasons. Observed changes in the metabolome could be linked to the clinical response to a study medication or any other kind of intervention. This could enable future predictions of drug efficacy or side effects based on the metabolome. Other potential benefits include a better understanding of an intervention’s mode of action. However, two questions are critical in ascertaining whether such an approach is feasible. The first question concerns the reproducibility of metabolite measurements. Metabolite levels in an individual need to be reasonably stable over time to allow for the measurement of changes elicited by an intervention. Few studies have investigated the reliability of metabolite concentrations across repeated measurements [1]–[3]. However, these are limited by a smaller number of metabolites analyzed. The second issue arises from the fact that almost all larger clinical trials are multicenter studies. To incorporate metabolomics into such studies, one has to validate realistic and cost effective ways of pre-analytic sample handling, such as 1) shipment, 2) choice of tube type and 3) repeated freeze-thaw cycles. To date, studies investigating sample stability during shipment focus on a small metabolite panel including cholesterol [4]–[6], vitamins [6] lipids [6], [7], amino acids [8], glucose [9] or acylcarnitines [10], or they are limited by a small sample size [11].In this study, we address questions regarding the reproducibility of targeted metabolomics measurements in the same individual at three different time points and of pre-analytic stability of metabolites.

## Materials and Methods

### Ethics Statement

All participants of this study gave written informed consent. The study was conducted according to the principles expressed in the Declaration of Helsinki and approved by the ethics committee of the Ludwig-Maximilians-University Munich (LMU), Germany (no. 086-06).

### Sample Collection and Preparation

Blood samples were collected from 22 healthy volunteers (5 men and 17 women), with a mean age of 30 (range: 22–52) after an overnight fast. Gender was found to be no confounder in this study. Information regarding medication and the last meal before each fasting period was collected for each sampling day. All participants were non-smokers. On day one, blood was taken from 20 participants (5 men and 15 women) in five 7.5 mL safety-monovettes (Sarstedt, Nümbrecht). For preparation of plasma (‘plasma-direct’), the K^+^EDTA^–^monovette was centrifuged directly (2000×g, 10 min). One monovette for serum preparation (‘serum W’ with clot activator) was centrifuged after 30 min of coagulation at room temperature (RT) (∼21°C). Serum W and plasma-direct samples were stored as 0.25 mL aliquots on dry ice and frozen at −80°C before measurement. The other three serum tubes (serum gel-barrier tubes with clot activator) were stored on cool packs (CP) (∼4°C) for 3, 6 and 24 h before centrifugation. Aliquots of 0.25 mL were stored at −80°C before measurement.

On day two, blood was taken from the same 20 participants in six 7.5 mL tubes. One tube for serum (serum W) and one for plasma (plasma-direct) were prepared as described above. The remaining four plasma tubes from each person were stored on CP (∼4°C) for 3, 6 and 24 h and one tube at RT (∼21°C) for 24 h before centrifugation. Aliquots of 0.25 mL plasma were stored at −80°C before measurement.

On day three, blood was collected from the same 20 participants in two 7.5 mL tubes for serum W and plasma-direct preparation (see above). For a subgroup of 13 volunteers (4 men and 9 women), blood was collected in three 7.5 mL serum gel-barrier tubes. One serum gel-barrier tube from each person was frozen immediately at −20°C and stored at −80°C without additional thawing before measurement (for thawing and tube type experiments). Aliquots of 0.25 mL of the remaining two serum gel-barrier tubes were frozen at −20°C and thawed once and twice after one week, respectively before storage at −80°C. From five subjects of this subgroup a second set of serum gel and serum W samples was collected for the thawing and tube type experiments during an additional collection day (1 men, 4 women). Additionally, 30 mL blood was taken from two different participants (2 women) in four 7.5 mL tubes, one for serum W and three for serum gel preparation (thawing and tube type experiments).

### Metabolite Analysis

The targeted metabolomics approach was based on electrospray ionization liquid chromatography–mass spectrometry (ESI-LC-MS/MS) and MS/MS measurements using the Absolute*IDQ*™ p180 kit (BIOCRATES Life Sciences AG, Innsbruck, Austria). The assay allows simultaneous quantification of 188 metabolites out of 10 µL plasma, including free carnitine, 40 acylcarnitines (Cx:y), 21 amino acids (19 proteinogenic amino acids, citrulline and ornithine), 21 biogenic amines, hexose (sum of hexoses – about 90–95% glucose), 90 glycerophospholipids (14 lysophosphatidylcholines (lysoPC) and 76 phosphatidylcholines (PC diacyl (aa) and acyl-alkyl (ae)), and 15 sphingolipids (SMx:y). The abbreviation Cx:y is used to describe the total number of carbons and double bonds of all chains, respectively (for more details, see [12]). The method of the Absolute*IDQ*™ p180 kit has been proven to be in conformance with the FDA-Guideline “Guidance for Industry - Bioanalytical Method Validation (May 2001”) [13], which implies proof of reproducibility within a given error range. Measurements were performed as described in the manufacturer’s manual UM-P180. The assay procedures of the Absolute*IDQ*™ p180 kit as well as the metabolite nomenclature have been described in detail previously [12], [14]. Sample handling was performed by a Hamilton Microlab STAR™ robot (Hamilton Bonaduz AG, Bonaduz, Switzerland) and an Ultravap nitrogen evaporator (Porvair Sciences, Leatherhead, U.K.). Mass spectrometric (MS) analyses were carried out on an API 4000 LC-MS/MS System (AB Sciex Deutschland GmbH, Darmstadt, Germany) equipped with 1200 Series HPLC (Agilent Technologies Deutschland GmbH, Boeblingen, Germany) and HTC PAL auto sampler (CTC Analytics, Zwingen, Switzerland) controlled by the Analyst 1.5.1 software. For the calculation of metabolite concentrations, internal standards served as a reference. Concentrations of all metabolites were calculated in µM. The analytical variance was determined by measuring metabolite concentrations of a reference sample of pooled human plasma with four to six replicates on each of the six plates.

### Statistical Analysis

All statistical analyses were performed with the statistic platform R, version 2.14.1 [15]. 29 metabolites were excluded from further analysis as they did not pass quality control (with more than 12% of measurements at zero concentration or missing and concentrations below LOD in more than 95% of all samples). 16 metabolites had a coefficient of variance (CV) across all plates above 25% in reference samples (see table S1). These were not excluded but labeled (*) in the tables and figures, as these metabolites may have a lower CV across all plates in future investigations.

Between and within-subject CVs (BCV and WCV) and between and within-plate CVs were calculated as the square root of the between- and within-subject/plate variance as determined using random effects models (R package *nlme*) divided by the mean metabolite concentration. The median within-plate CV was 9.31% and the median between-plate CV was 1.74E-4% (table S2). Reliability was expressed by the intraclass correlation coefficient (ICC (1)), calculated as between-subject variance divided by the sum of between- and within-subject variance [16]. Bias corrected and accelerated bootstrap confidence intervals were calculated for between- and within-subject CVs and ICC (1) using 10,000 bootstrap samples [17].

In order to explore time and temperature effects during simulation of transport in serum and plasma samples and the effect of tube type and number of thawing cycles on metabolite concentration in serum samples, nonparametric Friedman tests were performed [18]. Significant changes in concentration during the Friedman tests were afterwards tested in a pairwise manner against the reference (0 h) with Wilcoxon signed-rank tests. Similarly, significant differences in metabolite concentrations between serum and plasma baseline samples (serum W and plasma-direct) were tested in a pairwise manner using Wilcoxon signed-rank tests. For all performed tests the level of statistical significance was set at p<0.01.

A power analysis was performed for each metabolite for selected settings. The minimum concentration change between two paired settings (e.g. 0 h and 24 h) was calculated, for which the present study had a power of 80% to recognize it as significant, at the given sample size of 20 and at an alpha level of 0.01. To account for the reduced power of the non-parametric Wilcoxon signed-rank as compared to a parametric paired t-test, an asymptotic relative efficiency of 0.955 was assumed [19]. Results are given in table S3.

## Results

### Reliability of Serum and Plasma Metabolite Concentrations

**Figure 1:**
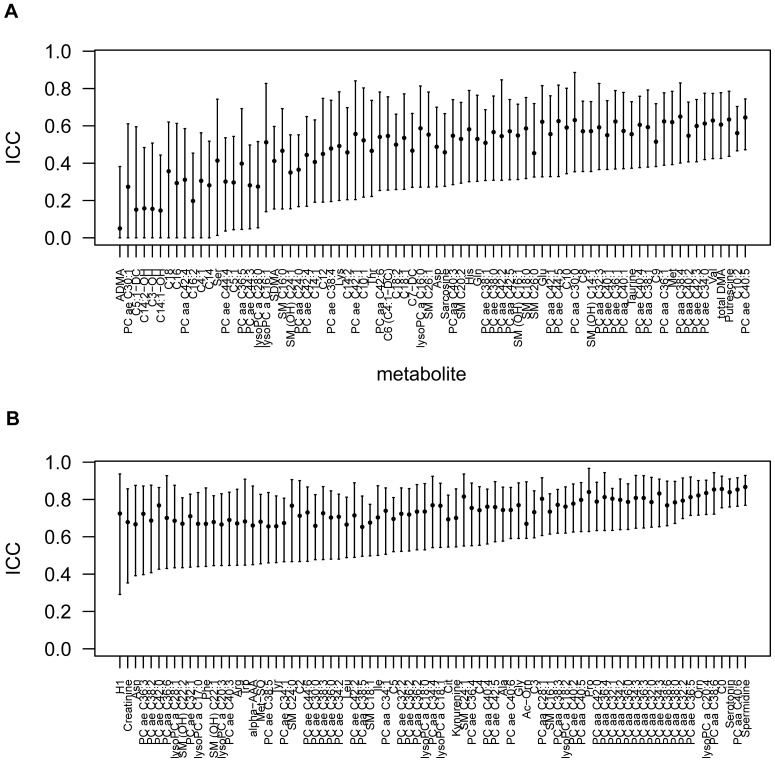
Median ICC with confidence intervals of serum metabolites. (A) Metabolites with median ICC-values below 0.65 and (B) metabolites with median ICC values above 0.65 are displayed.

**Figure 2:**
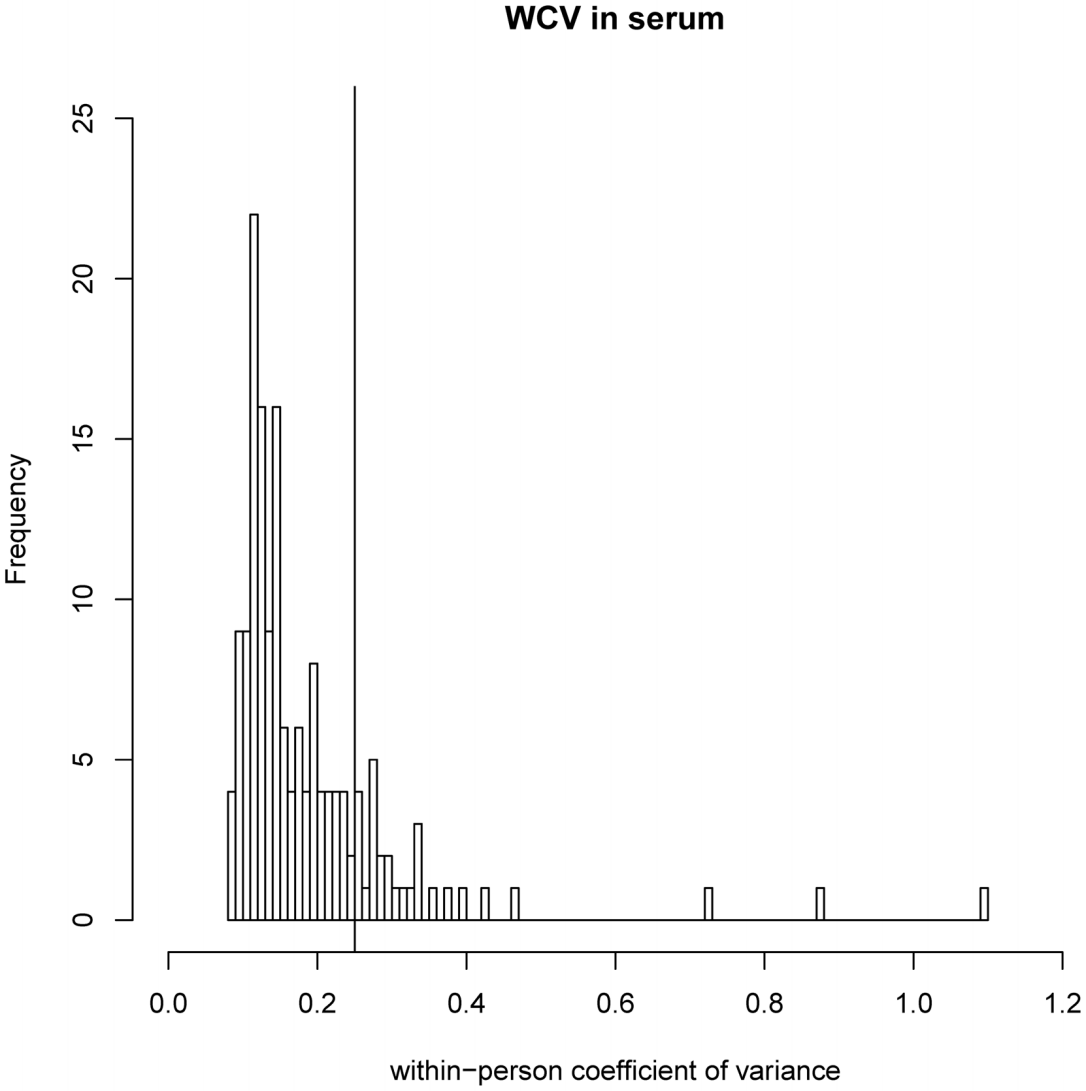
Histogram of within-subject coefficient of variance (WCV) in serum with mark at CV = 0.25.

Samples taken from 20 healthy human subjects on three different days were used to calculate the intraclass correlation coefficient (ICC), the within-person CV (WCV) and the between-person CV (BCV) for 159 metabolites in serum (figure 1) and plasma (figure S1). Table S4 summarizes results for ICC, WCV and BCV. In serum and plasma metabolites, 80% of WCV and 70% of BCV values were below 0.25 (figure 2, figure S2). In serum, the median WCV was 0.15 (range: 0.08–1.09) and the median BCV 0.20 (range: 0.05–1.56). Median WCV values of metabolite subclasses were concordant in serum and plasma samples, whereas median BCV values were slightly higher in serum samples. Both within- and between-person variance contribute to the ICC. Accordingly, the median ICC was higher in serum as compared to plasma samples.

The median ICC in plasma was 0.63. The lowest reliability in plasma was detected for PC ae C30∶1* (ICC = 2.28E-09, confidence interval (CI): 0.00–0.47) and the highest for creatinine (ICC = 0.90, CI: 0.84–0.95). In serum samples, the median ICC was 0.66. The lowest reliability in serum was observed for asymmetric dimethylarginine (ADMA) (ICC = 0.05, CI: 0.00–0.38) and the highest for spermidine* (ICC = 0.87, CI: 0.77–0.93). Regarding metabolite subclasses, reliability was lowest for acylcarnitines both in serum and plasma, as hydroxyacylcarnitines showed low reliability due to low concentrations. Long chain (>C10) and unsaturated acylcarnitines showed low reliability either due to low concentration or due to higher within-person variability compared to between-person variability. All other subclasses showed good (ICC >0.50) reliability. 101 metabolite concentrations were significantly higher in serum compared to plasma, comparing the median values of the baseline samples that were collected on three different days. Only sarcosine showed significantly lower concentrations in serum compared to plasma samples (table S5). There was no evidence of any effects of gender and last meal composition before each overnight fasting on metabolite concentrations (data not shown).

### Stability of Metabolites during Shipment Simulation

#### Simulated shipment on cool packs and at room temperature

We simulated different ways of sample collection and shipment that might occur in multicenter clinical trials: Transport of non-centrifuged samples on cool packs (CP) within 3, 6 and 24 hours (plasma and serum) or at room temperature (RT) for 24 h (plasma only).

**Figure 3:**
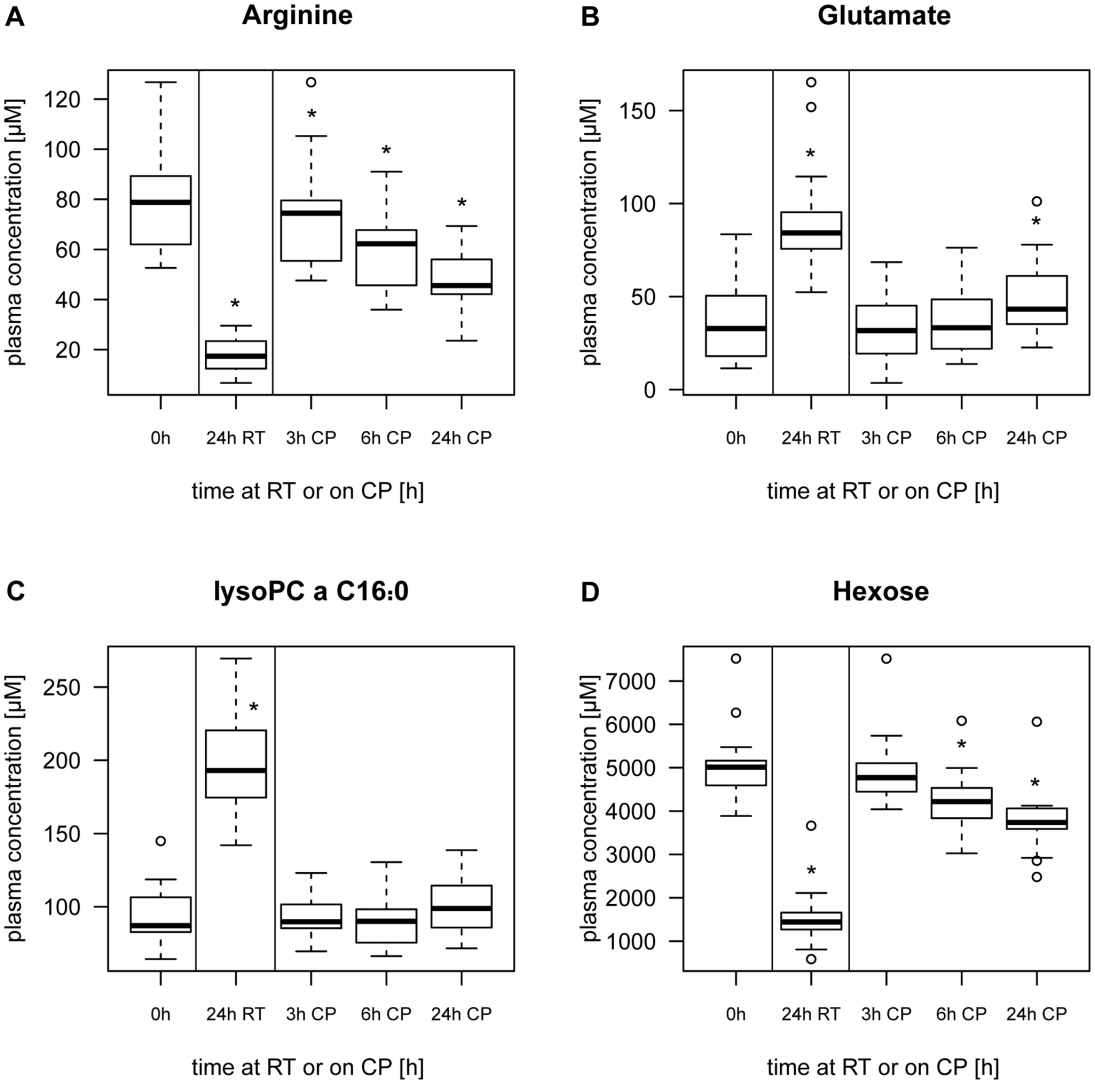
Stability of metabolites in plasma during shipment simulation. Example of (A), (D) decreasing and (B), (C) increasing metabolite concentration of plasma samples at room temperature (RT) and on cool packs (CP). Stars in boxplots indicate significant difference in concentration compared to baseline (0 h). (Wilcoxon signed rank test, significance level p<0.01).

In plasma samples, 44 out of 159 metabolites showed significant concentration changes between baseline (0 h) and any other setting (table S6) as determined by Friedman tests (p<0.01). 145 plasma metabolite concentrations were stable for at least 24 h on CP and 115 of these were also stable for at least 24 h at RT. Of the 44 metabolites with significantly changed concentrations at RT, 35 showed increasing concentrations after 24 h (figures S3, S4, S5, S6, S7, S8, S9, S10, S11, and S12) whereas 9 showed decreasing concentrations, including C4∶1 (figure S4), arginine (figure 3), methionine (figure S6), serotonin* (figure S9), four PC aa’s (PC aa C30∶0, PC aa C32∶1, PC aa C32∶2 and PC aa C34∶3, see figure S12) and hexose (figure 3). Figure 3 shows examples of significantly altered metabolite concentrations in plasma.

The compound class of amino acids was the least stable metabolite class. In the compound class of glycerophospholipids, all metabolites had stable concentrations on CP for at least 24 h. Concentrations in 2 out of 10 lysoPCs (lysoPC a C28∶0 and lysoPC a C28∶1) were even stable at RT for 24 h. 32 of 36 PC aa concentrations and all PC ae concentrations remained stable at RT. The compound class of sphingolipids was the most stable one, as all analyzed sphingolipid concentrations were stable on CP and at RT for at least 24 h.

**Figure 4:**
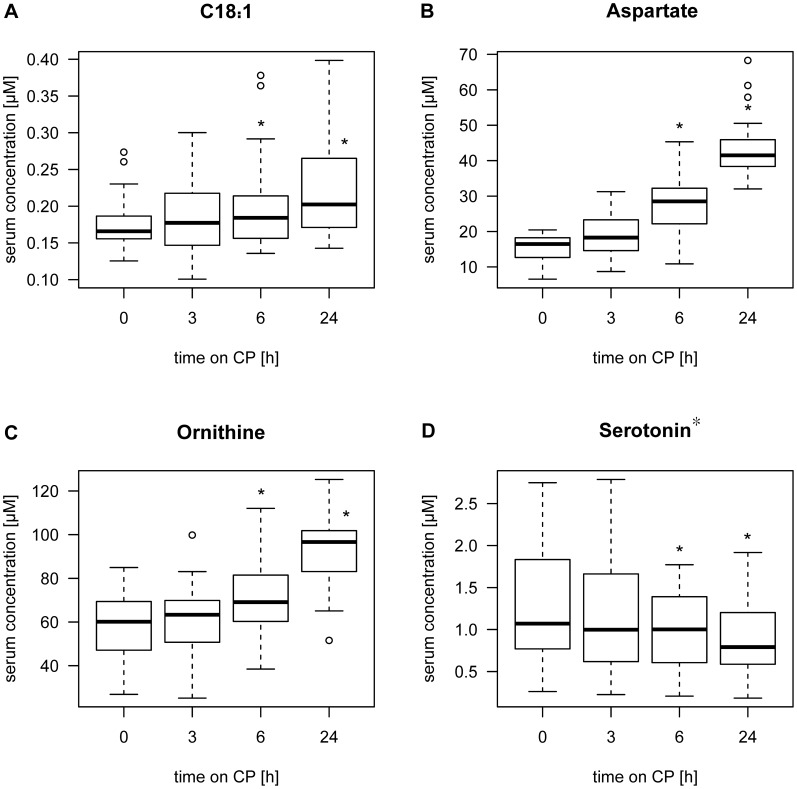
Stability of metabolites in serum during shipment simulation. Example of (A)-(C) increasing and (D) decreasing metabolite concentration during transportation simulation of serum samples on cool packs (CP). Stars in boxplots indicate significant difference in concentration compared to baseline (0 h). (Wilcoxon signed rank test, significance level p<0.01).

All significantly increased or decreased metabolite concentrations during shipment simulation in serum showed similar tendencies in plasma samples over time on CP (see figures S3, S4, S5, S6, S7, S8, S9, S10, S11, S12, S13, S14, S15, S16, and S17). In plasma, a larger number of metabolites had stable concentrations for 24 h on CP as compared to serum. In total, 140 of 159 serum metabolites had stable concentrations on CP for at least 24 h. 19 metabolites showed significant changes in serum concentration during storage on CP within the first 24 h (table 1). 14 metabolite concentrations increased significantly with time (figure S13, S14, S15, S16, and 17), whereas five metabolite concentrations decreased significantly (arginine, putrescine*, serotonin*, spermidine* and hexose). Figure 4 shows examples of altered metabolite concentrations in serum. In concordance with plasma samples, the group of amino acids was the least stable one in serum metabolites.

**Table 1:** Impact of transportation simulation on metabolite concentrations in serum samples.

Metabolite	p-value(Friedman)	Acceptable delaytime on cool packs
C18∶1	1.92E-04	3 h
C18∶2	2.52E-03	6 h
Arginine	1.52E-04	3 h
Asparagine	1.49E-06	6 h
Aspartate	6.08E-11	3 h
Glutamate	1.20E-10	0 h
Glycine	4.79E-05	6 h
Leucine	1.01E-03	6 h
Lysine	8.49E-04	6 h
Ornithine	1.18E-09	3 h
Phenylalanine	2.69E-05	6 h
Serine	9.04E-07	6 h
Threonine	1.86E-03	6 h
Putrescine*	1.21E-06	0 h
Sarcosine	1.82E-03	6 h
Serotonin*	2.67E-03	3 h
Spermidine*	4.17E-04	0 h
Taurine	1.00E-07	3 h
Hexose	6.13E-07	3 h

* Metabolites with coefficient of variance across all plates above 25% in reference samples.

Metabolites that showed significant changes in serum concentration on cool packs for 3, 6 or 24 h compared to baseline (0 h) (Friedman test, p<0.01) and acceptable delay time for each metabolite during transportation (Wilcoxon signed rank test, p<0.01).

#### Effect of tube type on serum metabolites

In multicenter studies, the use of serum gel-barrier tubes is preferred due to the easy handling at the blood collection site and persistent separation of serum during shipment of tubes. In this study, possible interactions of the gel with the metabolites in human serum samples were analyzed. Blood was drawn into serum gel-barrier tubes and serum W tubes with clotting activator only (control). After centrifugation, whole gel-barrier tubes and separated serum from serum W tubes were frozen until analysis.

**Figure 5:**
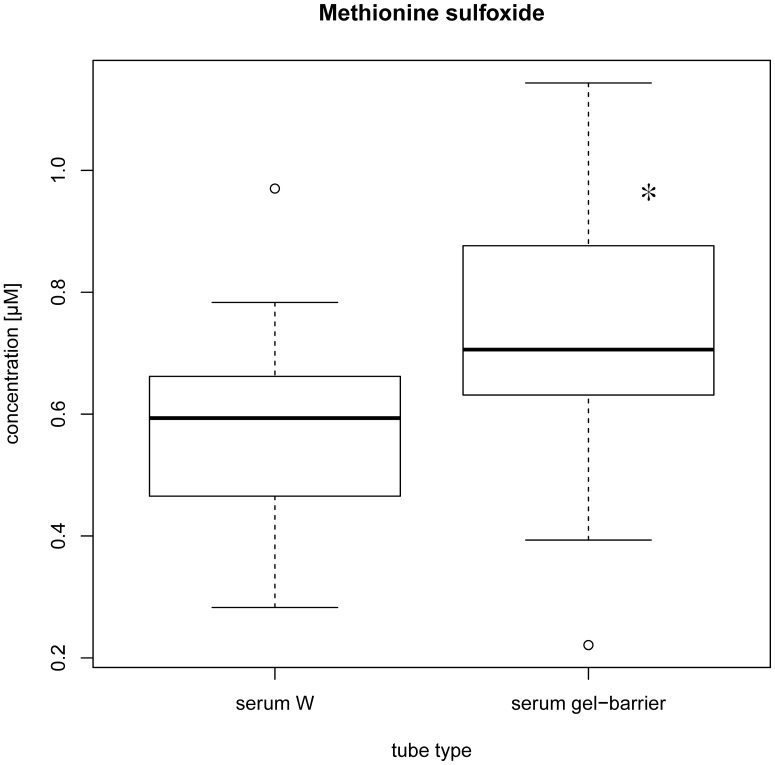
Effect of tube type on serum metabolites. Stars in boxplots indicate significant differences in concentration between methionine sulfoxide in serum W tubes with clotting activator and serum gel-barrier tubes. (Friedman test, significance level p<0.01).

Methionine sulfoxide showed significantly increased concentrations in serum gel-barrier tubes as compared to serum W (see figure 5). For all other investigated metabolites, serum gel-barrier tubes had no significant effect on concentration, compared to serum W tubes.

#### Effect of freeze-thaw cycles on serum metabolites

Repeated freezing and thawing of samples is sometimes inevitable due to a limited number of aliquots. When serum samples were subjected to two freeze-thaw cycles, most metabolite concentrations were stable. Except methionine sulfoxide, all 159 investigated metabolites of this panel maintained stable concentrations after one freeze-thaw cycle. Only eleven out of 159 metabolites revealed significantly decreased concentrations after two freeze-thaw cycles, including C10∶1, three amino acids (isoleucine, tryptophane and valine), five phosphatidylcholines (PC aa C32∶2, PC ae C36∶2, PC ae C36∶5, PC ae C40∶1 and PC ae C42∶0), acetylornithine and SM C16∶0 (table S7).

#### Power calculation

The minimal effect size (i.e. absolute difference in metabolite concentration) that could be detected for each metabolite with a given power of 0.8 was calculated for selected sample pairs. table S3 gives estimates of detectable effect sizes for individual metabolites in our study. This information should be informative for planning future studies. This study was not powered to significantly detect smaller effects than those displayed.

## Discussion

### Reliability of Serum and Plasma Metabolite Concentrations

The analysis of three fasting blood samples within a 14 day period showed that most of the 159 metabolite levels remained stable within the same individual. In serum and plasma metabolites, 80% of WCV and 70% of BCV values were below 0.25 and showed good reliability within individuals, independent of the characteristics of the study population. An ICC above 0.50 has been defined as good reliability [2]. In our study, more than 75% of metabolites in serum and plasma samples fulfilled this criterion. Compared to a population-based approach, our ICC values were artificially low due to the homogeneous study cohort. Metabolites with good reliability included saturated short- and medium acylcarnitines, amino acids, biogenic amines, glycerophospholipids, sphingolipids and hexose. These findings are in line with Floegel *et al*. [2].

Creatinine was found to have the lowest WCV and highest reliability in plasma. In muscle cells, the ring closing of creatine forms creatinine in a chemical equilibrium [20]. As the quantity of creatine per unit of skeletal muscle and the reaction to creatinine is constant [21], the produced amount of creatinine is dependent on the muscle mass. Concentration of creatinine is therefore constant and within-person variability is relatively low in healthy people, as long as the muscle mass remains constant.

Reliability of metabolites was slightly higher in serum compared to plasma. 101 metabolite concentrations were significantly higher in serum samples compared to plasma. 86 of these metabolites showed similar results in Yu *et al*. Two of the 101 metabolites (C10∶2 and PC aa C40∶2) were excluded by Yu *et al.* and 12 of these 101 metabolites were not part of the metabolite panel of Yu *et al*. In our study, SM (OH) C24∶1 showed significantly higher concentrations in serum compared to plasma. This finding is not in line with Yu *et al*. [22], [23]. Ladenson *et al.* found a 5% lower glucose concentration in plasma compared to serum [23]. Although glucose concentration was lower in plasma samples, the difference between serum and plasma levels of glucose was not significant in our study. We found no effect of sex and last meal composition before overnight fasting in contrast to other studies [24]–[26]. This could be explained by the sample size and the unbalanced ratio of men to women.

Our study goes beyond previous investigations by using a broader metabolite panel and measuring three samples per individual at the same time, in a well-established and validated targeted metabolomics approach. The observed high reliability of amino acids was consistent with the results of Floegel *et al*. [2]. However, reliability for six additional amino acids was shown in our study. The good repeatability of phospholipid measurements in plasma samples observed by Ma *et al*. was in line with our observations [1]. Classification of reliability of acylcarnitines, glycerophospholipids, sphingolipids and hexose was concordant with that of Floegel *et al*. [1]. Widjaja *et al*. in concordance with our study showed that a single measurement of glucose is sufficient in plasma samples [3].

No analytical duplicates were measured. Therefore, it was not possible to further divide the total within-person variability into biological and analytical variability. However, regarding the between and within-plate CVs derived from repeated measurements of reference samples (table S2), analytical variance can be assumed to be low. Only samples of healthy subjects were analyzed, therefore variability in non-healthy patients could vary from these results.

In sum, we found that most metabolite concentrations in a targeted approach are highly stable over time in a fasted individual. The WCVs obtained in this study can help to judge whether single measurements of a metabolite are sufficient to describe differences between groups of human subjects. They can also assist with the interpretation of the effects of a medication or another intervention on an individual’s serum or plasma metabolome.

### Stability of Metabolites during Shipment Simulation

#### Simulated shipment on cool packs and at room temperature

The majority of the analyzed metabolites had stable concentrations for at least 24 h on CP (plasma and serum) and at RT (plasma). In plasma and serum samples, no significant changes in concentration during transportation for 24 h on CP and at RT were detected for PC ae’s and sphingolipids. The compound class of amino acids was most fragile during simulated transportation. Plasma and serum metabolite stability showed good coherence.

Within the group of acylcarnitines, Yang *et al.* showed decreasing concentrations of C8∶0, C18∶1 and C18∶2 and stable concentrations of carnitine and eight other acylcarnitines (C2, C3, C4, C5, C6, C14, C16, C16∶1) after 24 h at 37°C. The stability of carnitine, C2, C3, C4 and C5 for 24 h at 37°C is in line with our results (24°h at RT), whereas C18∶1 and C18∶2 showed an opposing trend. Stability of C14 and C16 was not confirmed in our study. In contrast to Yang *et al.*, our results showed stability of C8 for 24 h at RT. The reason for these partly contradictory results could be the small size of three subjects in Yang *et al.* and the different origin of specimen (rat in Yang *et al.*) [11]. LC-MS measurement of 18 amino acids in porcine cerebrospinal fluid, stored at RT for up to 2 h, showed increased concentrations in ten amino acids (alanine, asparagine, glycine, glutamate, histidine, isoleucine, phenylalanine, serine, threonine, tyrosine), which is in line with our observations, except that glycine concentration is stable in our experiments [8]. Yang *et al*. showed stability of nine amino acids for up to 24 h at 37°C in rat plasma. [11]. Only valine and tryptophane stability is in accordance with our results in human plasma samples. The decreasing levels of four PC aa’s and increasing levels of eight lysoPCs during storage at RT could be explained by the hydrolysis of phosphatidylcholine to yield a lysophospholipid and free fatty acid, catalyzed by a phospholipase [27]. This result shows remaining activity of phospholipase in non-centrifuged plasma samples at RT but not at 4°C. Increasing lysoPC levels at RT were also observed in Yang *et al*. [11]. Sphingomyelins, which were found to play an important role in β-cell function [28], were the most stable compound class both in plasma and serum samples in this study. This finding indicates low enzymatic activity of sphingomyeline degrading and synthesizing enzymes at 4°C and RT. Decreasing hexose concentration during storage due to glycolysis, especially at RT, is consistent with the literature [9].

#### Effect of tube type on serum metabolites

The use of serum gel tubes was investigated by comparing samples drawn in serum W tubes and serum gel-barriertubes. Only methionine sulfoxide showed significant changes in concentration in gel-barrier tubes compared to serum W tubes. This indicates a possible interaction of the gel with the investigated metabolite. Thus, based on our data, the use of serum gel-barrier tubes should be avoided if the measurement of methionine sulfoxide is of interest. However, the use of serum gel-barrier tubes is preferred for all other analyzed metabolites in multicenter settings due to the advantages for sample handling.

#### Effect of freeze-thaw cycles on serum metabolites

Except methionine sulfoxide, all 159 investigated metabolites of this panel had stable concentrations after one freeze-thaw cycle. Only eleven metabolites showed significantly decreased concentrations after two freeze-thaw cycles. Targeted and non-targeted analyses have investigated the stability of several serum and plasma metabolites in repeated freeze-thawing experiments [29]–[31] and showed only minor changes in metabolite concentration due to thawing. Cuhadar *et al.* showed stability of creatinine and glucose in serum samples for up to ten freeze-thaw cycles, which is in line with our results [31]. In the study of Zivkovic *et al.* serum samples were exposed to one, two or three freeze-thaw cycles [30]. Concentrations of lysoPC a C16∶0 were significantly altered after two freeze-thaw cycles (p = 0.048), which is not in line with our results, as we used a significance level of p = 0.01. The stability of phosphatidylcholine PC 15∶0, PC 16∶0 and PC 18∶0 for up to three freeze-thaw cycles, which was shown by Zivkovic *et al.,* is in line with our results.

Based on our data, the optimal method of sample shipment in multicenter trials is to collect samples in serum in gel-barrier tubes, to centrifuge them on site and to ship the samples while frozen, as at higher temperatures some amino acids and biogenic amines are not stable in both serum and plasma. Shipment of non-processed serum or plasma results in instability of more metabolites, but can be a simple and cost-effective alternative for selected questions.

## Supporting Information

Figure S1 **Median ICC with confidence intervals of plasma metabolites.** (A) Metabolites with median ICC-values below 0.62 and (B) metabolites with median ICC values above 0.62 are displayed.
(TIFF)

Figure S2 **Histogram of WCV in plasma with mark at CV = 0.25.**
(TIFF)

Figure S3 **Changes in metabolite concentration during transportation simulation of plasma samples.** (A) C 10∶2, (B) C14*, (C) C14∶1-OH* and (D) C16. Stars in boxplots indicate significant difference in concentration compared to baseline (0 h). (Wilcoxon signed rank, significance level p<0.01).
(TIFF)

Figure S4 **Changes in metabolite concentration during transportation simulation of plasma samples.** (A) C18*, (B) C18∶1, (C) C18∶2 and (D) C4∶1. Stars in boxplots indicate significant difference in concentration compared to baseline (0 h). (Wilcoxon signed rank, significance level p<0.01).
(TIFF)

Figure S5 **Changes in metabolite concentration during transportation simulation of plasma samples.** (A) Alanine, (B) Asparagine, (C) Aspartate and (D) Histidine. Stars in boxplots indicate significant difference in concentration compared to baseline (0 h). (Wilcoxon signed rank, significance level p<0.01).
(TIFF)

Figure S6 **Changes in metabolite concentration during transportation simulation of plasma samples.** (A) Isoleucine, (B) Leucine, (C) Lysine and (D) Methionine. Stars in boxplots indicate significant difference in concentration compared to baseline (0 h). (Wilcoxon signed rank, significance level p<0.01).
(TIFF)

Figure S7 **Changes in metabolite concentration during transportation simulation of plasma samples.** (A) Ornithine, (B) Phenylalanine, (C) Proline and (D) Serine. Stars in boxplots indicate significant difference in concentration compared to baseline (0 h). (Wilcoxon signed rank, significance level p<0.01).
(TIFF)

Figure S8 **Changes in metabolite concentration during transportation simulation of plasma samples.** (A) Threonine, (B) Tyrosine, (C) Acetylornithine and (D) ADMA. Stars in boxplots indicate significant difference in concentration compared to baseline (0 h). (Wilcoxon signed rank, significance level p<0.01).
(TIFF)

Figure S9 **Changes in metabolite concentration during transportation simulation of plasma samples.** (A) alpha-AAA, (B) Serotonin*, (C) Spermidine* and (D) Taurine. Stars in boxplots indicate significant difference in concentration compared to baseline (0 h). (Wilcoxon signed rank, significance level p<0.01).
(TIFF)

Figure S10 **Changes in metabolite concentration during transportation simulation of plasma samples.** (A) total DMA, (B) lysoPC a C16∶1, (C) lysoPC a C17∶0 and (D) lysoPC a C18∶0. Stars in boxplots indicate significant difference in concentration compared to baseline (0 h). (Wilcoxon signed rank, significance level p<0.01).
(TIFF)

Figure S11 **Changes in metabolite concentration during transportation simulation of plasma samples.** (A) lysoPC a C18∶1, (B) lysoPC a C18∶2, (C) lysoPC a C20∶3 and (D) lysoPC a C20∶4. Stars in boxplots indicate significant difference in concentration compared to baseline (0 h). (Wilcoxon signed rank, significance level p<0.01).
(TIFF)

Figure S12 **Changes in metabolite concentration during transportation simulation of plasma samples.** (A) PC aa C30∶0, (B) PC aa C32∶1, (C) PC aa C32∶2 and (D) PC aa C34∶3. Stars in boxplots indicate significant difference in concentration compared to baseline (0 h). (Wilcoxon signed rank, significance level p<0.01).
(TIFF)

Figure S13 **Changes in metabolite concentration during transportation simulation of serum samples.** (A) C18∶1, (B) C18∶2, (C) Arginine and (D) Asparagine. Stars indicate significant difference in concentration compared to baseline (0 h). (Wilcoxon signed rank, significance level: p<0.01).
(TIFF)

Figure S14 **Changes in metabolite concentration during transportation simulation of serum samples.** (A) Aspartate, (B) Glutamate, (C) Glycine and (D) Leucine. Stars indicate significant difference in concentration compared to baseline (0 h). (Wilcoxon signed rank, significance level: p<0.01).
(TIFF)

Figure S15 **Changes in metabolite concentration during transportation simulation of serum samples.** (A) Lysine, (B) Ornithine, (C) Phenylalanine and (D) Serine. Stars indicate significant difference in concentration compared to baseline (0 h). (Wilcoxon signed rank, significance level: p<0.01).
(TIFF)

Figure S16 **Changes in metabolite concentration during transportation simulation of serum samples.** (A) Threonine, (B) Putrescine*, (C) Sarcosine and (D) Serotonin*. Stars indicate significant difference in concentration compared to baseline (0 h). (Wilcoxon signed rank, significance level: p<0.01).
(TIFF)

Figure S17 **Changes in metabolite concentration during transportation simulation of serum samples.** (A) Spermidine*, (B) Taurine and (C) Hexose. Stars indicate significant difference in concentration compared to baseline (0 h). (Wilcoxon signed rank, significance level: p<0.01).
(TIFF)

Table S1 **List of all metabolites.** Metabolites that did not pass quality control (#) and metabolites that had a coefficient of variance (CV) across all plates above 25% in reference samples (*) are marked appropriately.
(XLS)

Table S2 **Within-and between plates CV.** CVs are calculated on the basis of multiple reference samples on each plate.
(XLS)

Table S3 **Effect size and mean concentrations for each metabolite in plasma and serum samples.** Given are minimum absolute differences in concentration [µM] for each metabolite in selected settings that are detectable given the sample size of 20, the observed variance in metabolite concentration changes, a power of 0.8 and a significance level of 0.01. Mean metabolite concentrations [µM] for plasma-direct and serum gel-barrier (0× thawed) are given as a comparison.
(XLS)

Table S4 **ICC(1), WCV and BCV with confidence intervals (CI) in serum and plasma metabolites.**
(XLS)

Table S5 **Comparison of the median metabolite concentrations in serum and plasma samples.** Serum W and plasma-direct baseline samples that were collected on three days within 20 subjects were tested in a pairwise manner using wilcoxon-signed rank test (p<0.01).
(XLS)

Table S6 **Impact of transportation simulation on metabolite concentrations in plasma samples.** Changes in concentration after 3, 6 or 24 h on CP or at RT after 24 h and acceptable delay time for each metabolite during transportation (Wilcoxon signed rank test, comparison with baseline, significance level p<0.01).
(XLS)

Table S7 **Significant changes in serum metabolite concentrations due to freeze-thawing cycles.** Overall significant changes in metabolite concentrations in serum gel tubes (Friedman test, significance level p<0.01) and changes after one and two freeze-thaw cycles, each one tested against serum gel control (Wilcoxon signed rank, significance level p<0.01).
(XLS)
