# Supplementary figures and images for: Targeted Metabolomics Identifies Reliable and Stable Metabolites in Human Serum and Plasma Samples
Source: PLoS One. 2014 Feb 24;9(2):e89728. doi: 10.1371/journal.pone.0089728 (PMC3933650; doi:10.1371/journal.pone.0089728)

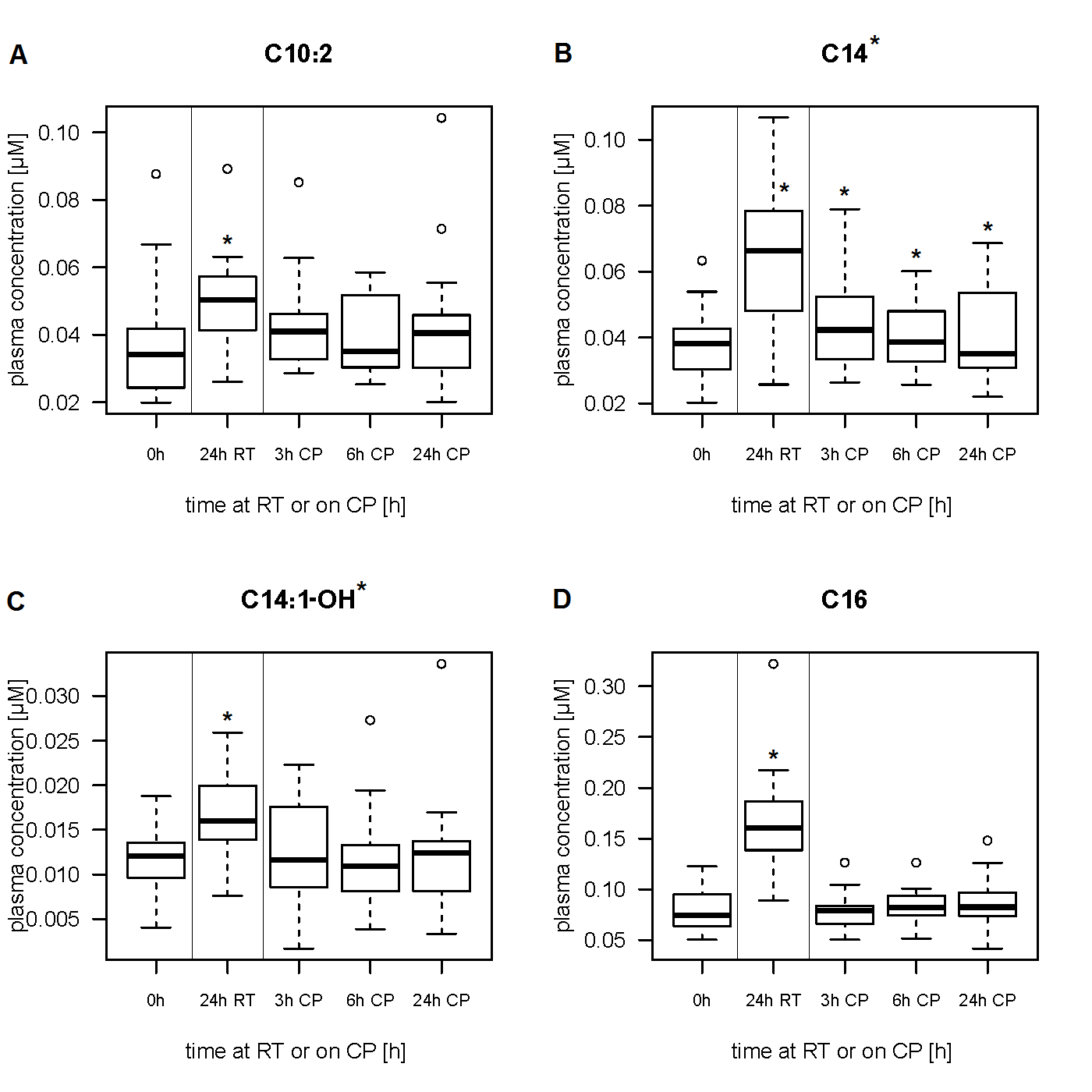

Supplement: Figure S3 — Changes in metabolite concentration during transportation simulation of plasma samples. (A) C 10∶2, (B) C14*, (C) C14∶1-OH* and (D) C16. Stars in boxplots indicate significant difference in concentration compared to baseline (0 h). (Wilcoxon signed rank, significance level p<0.01). (TIFF) [file pone.0089728.s003.tiff]

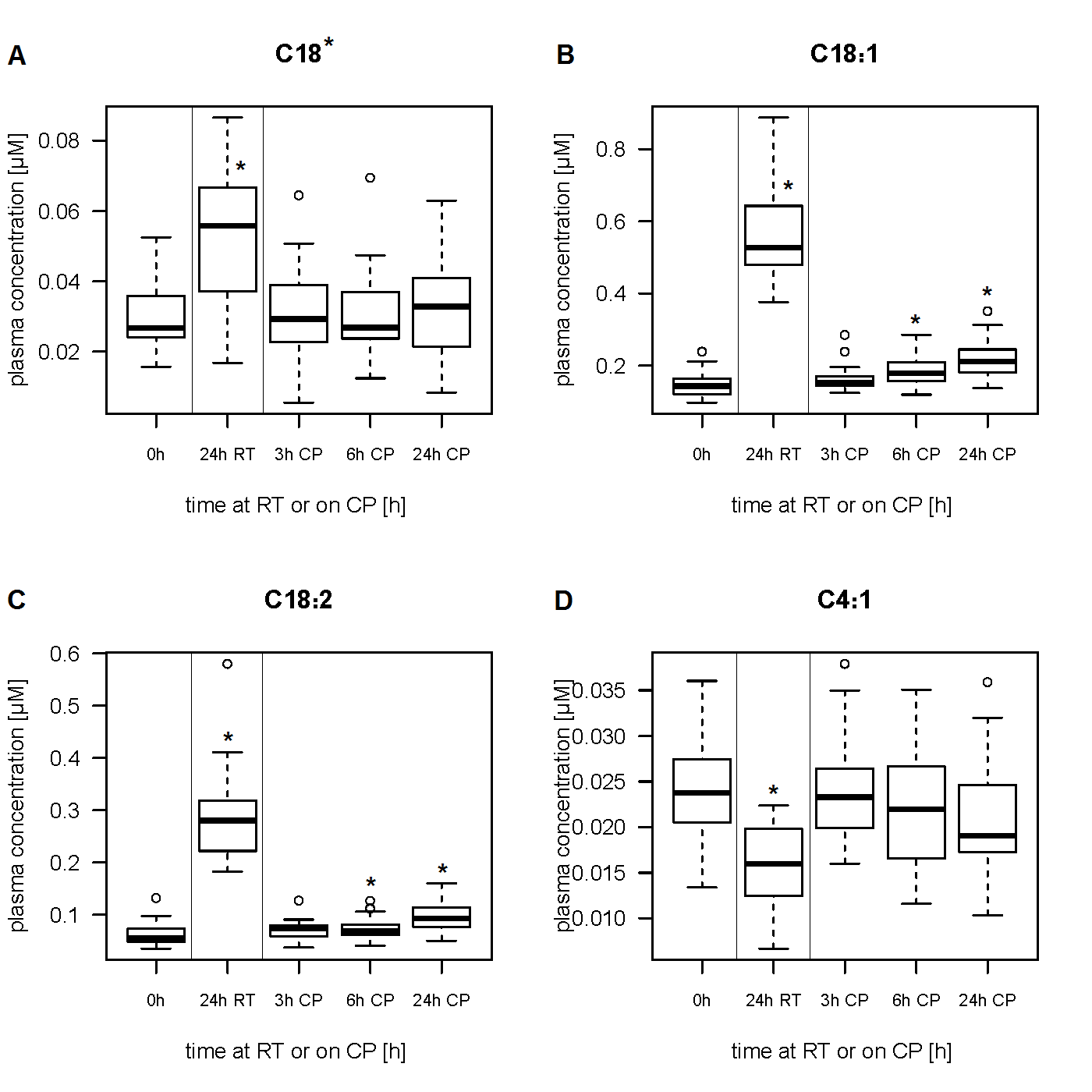

Supplement: Figure S4 — Changes in metabolite concentration during transportation simulation of plasma samples. (A) C18*, (B) C18∶1, (C) C18∶2 and (D) C4∶1. Stars in boxplots indicate significant difference in concentration compared to baseline (0 h). (Wilcoxon signed rank, significance level p<0.01). (TIFF) [file pone.0089728.s004.tiff]

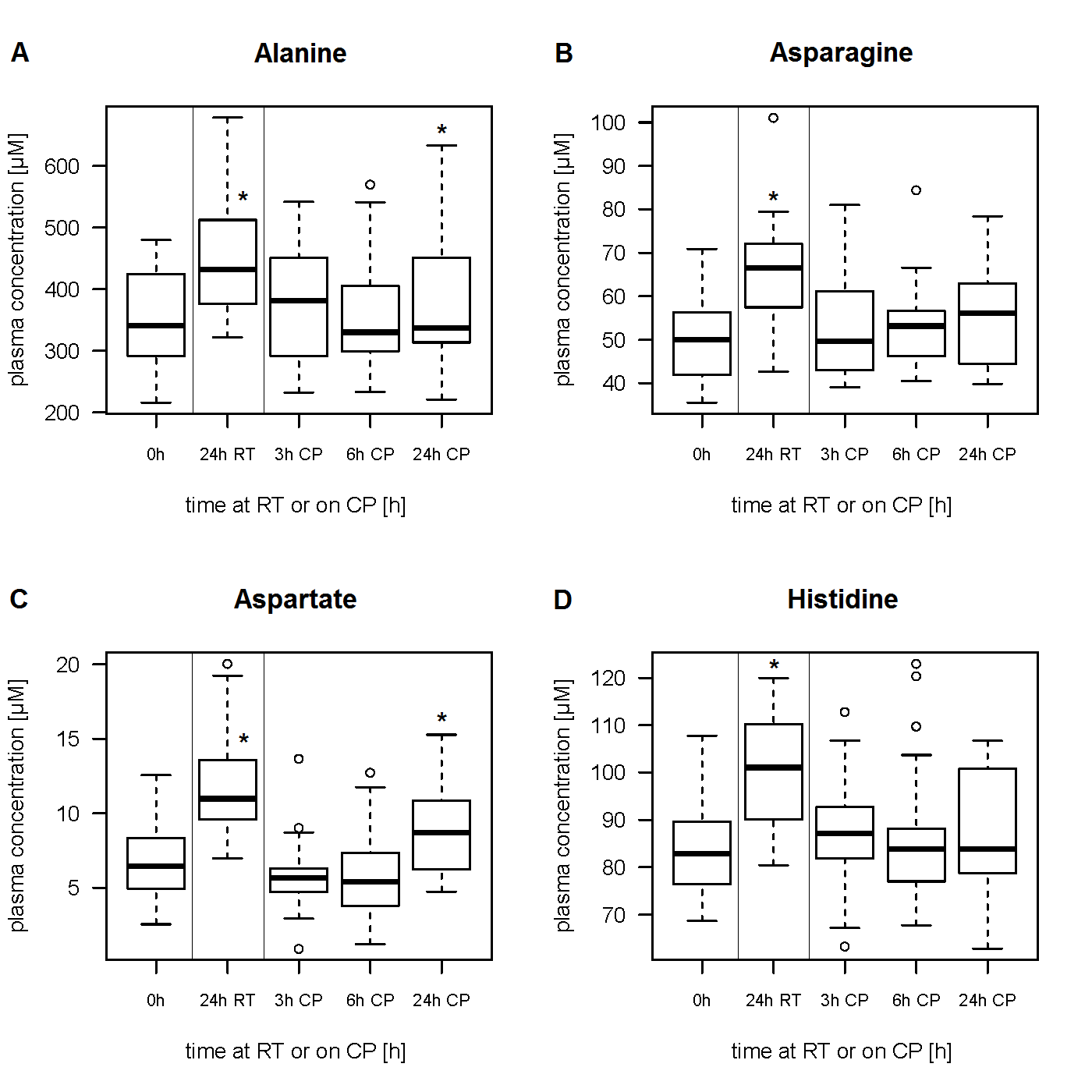

Supplement: Figure S5 — Changes in metabolite concentration during transportation simulation of plasma samples. (A) Alanine, (B) Asparagine, (C) Aspartate and (D) Histidine. Stars in boxplots indicate significant difference in concentration compared to baseline (0 h). (Wilcoxon signed rank, significance level p<0.01). (TIFF) [file pone.0089728.s005.tiff]

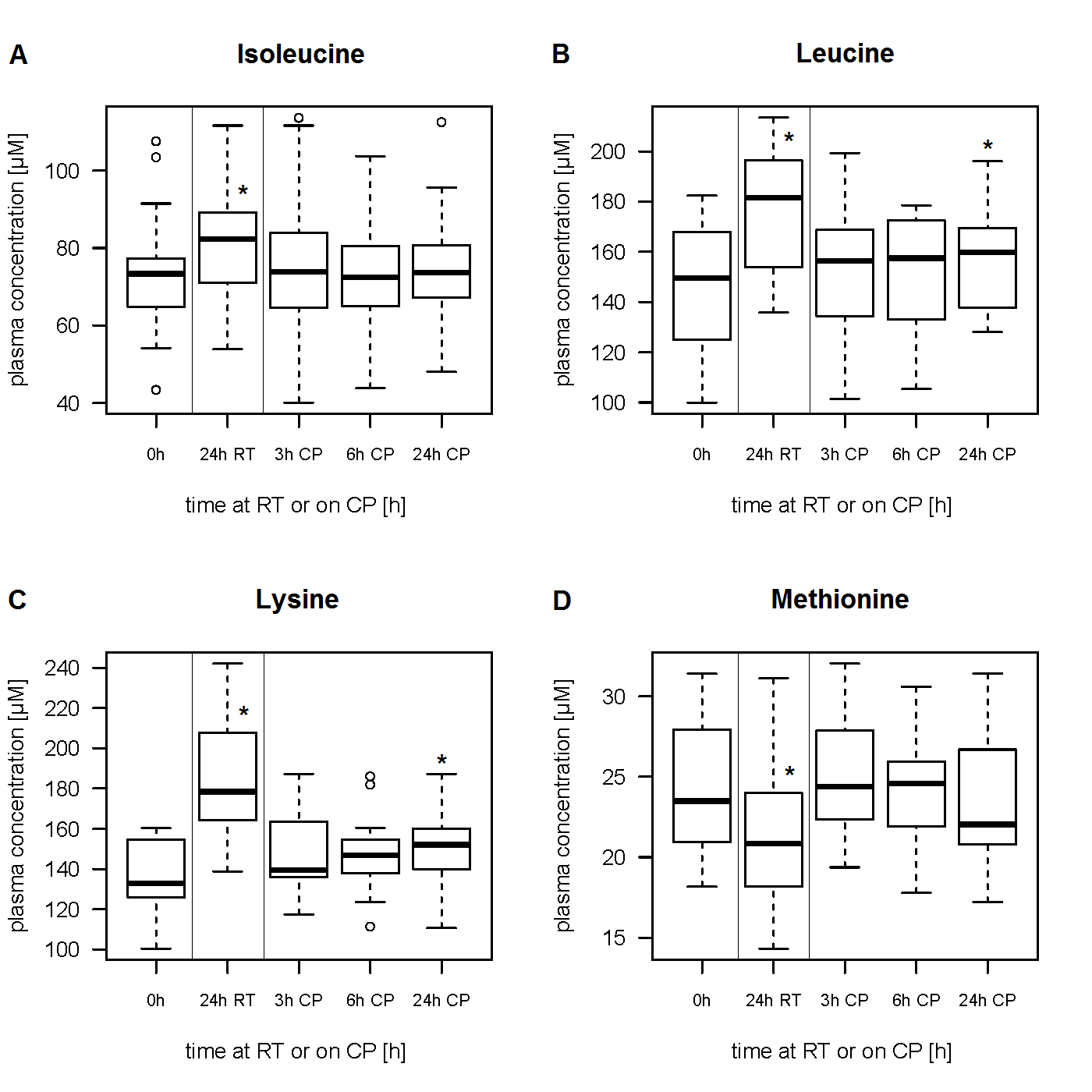

Supplement: Figure S6 — Changes in metabolite concentration during transportation simulation of plasma samples. (A) Isoleucine, (B) Leucine, (C) Lysine and (D) Methionine. Stars in boxplots indicate significant difference in concentration compared to baseline (0 h). (Wilcoxon signed rank, significance level p<0.01). (TIFF) [file pone.0089728.s006.tiff]

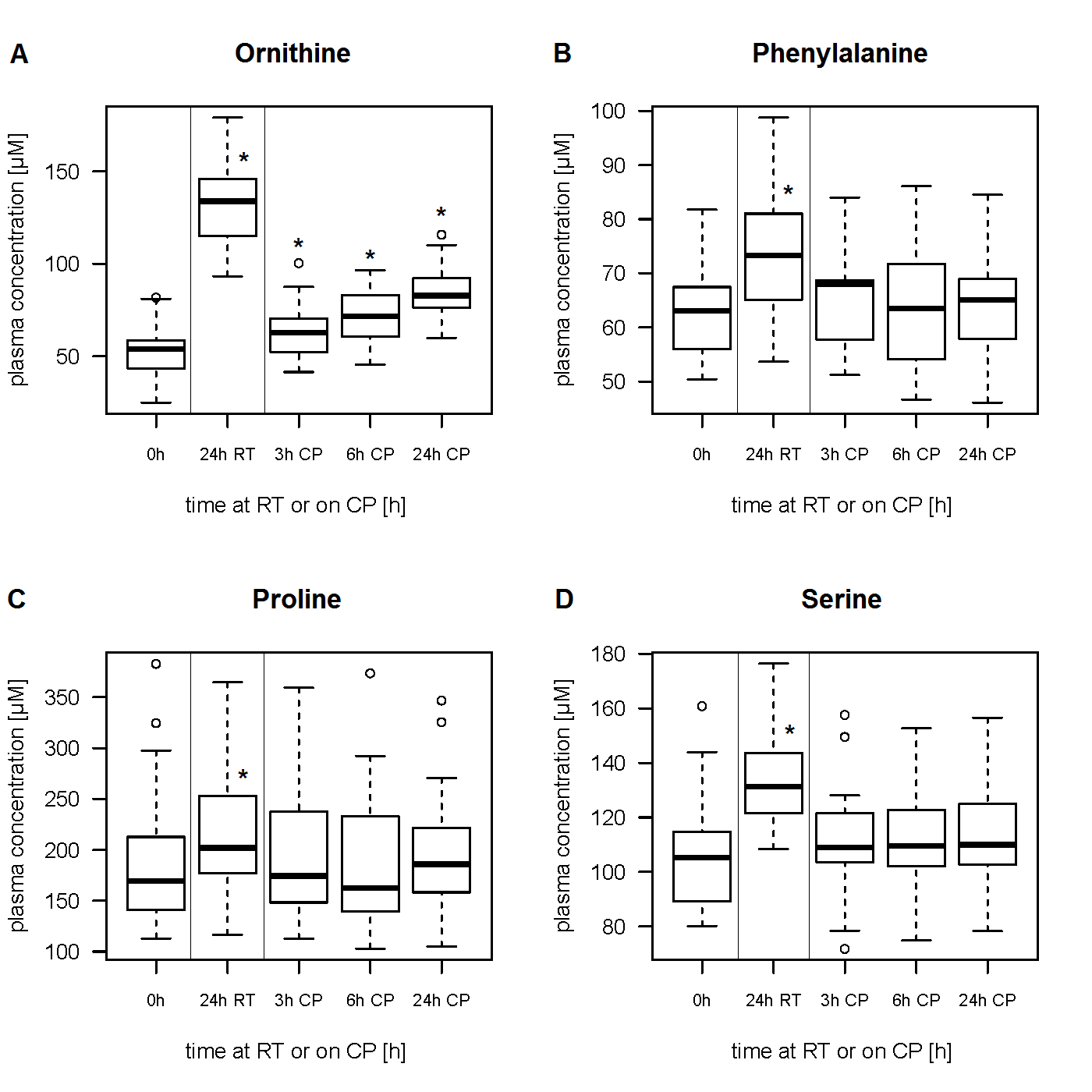

Supplement: Figure S7 — Changes in metabolite concentration during transportation simulation of plasma samples. (A) Ornithine, (B) Phenylalanine, (C) Proline and (D) Serine. Stars in boxplots indicate significant difference in concentration compared to baseline (0 h). (Wilcoxon signed rank, significance level p<0.01). (TIFF) [file pone.0089728.s007.tiff]

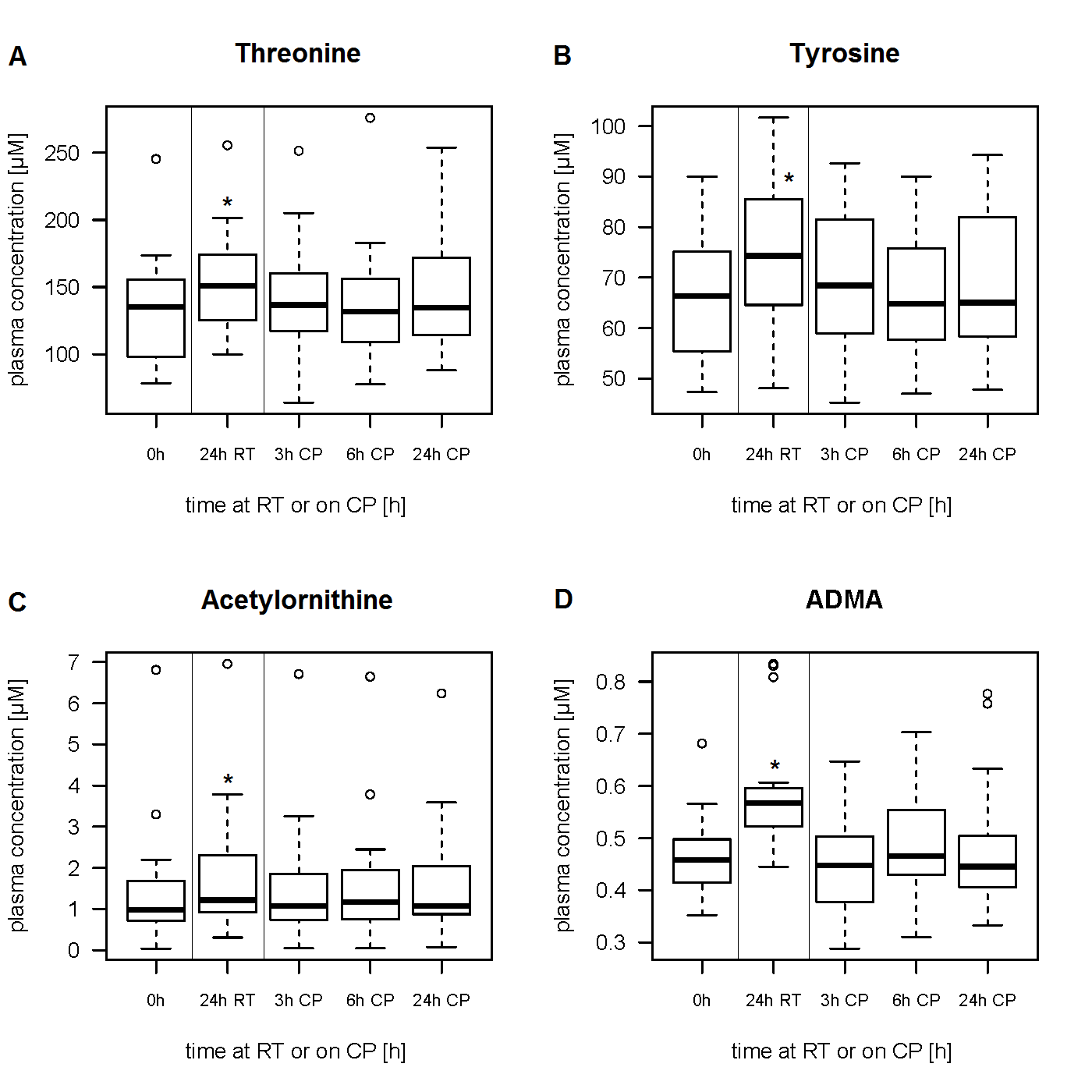

Supplement: Figure S8 — Changes in metabolite concentration during transportation simulation of plasma samples. (A) Threonine, (B) Tyrosine, (C) Acetylornithine and (D) ADMA. Stars in boxplots indicate significant difference in concentration compared to baseline (0 h). (Wilcoxon signed rank, significance level p<0.01). (TIFF) [file pone.0089728.s008.tiff]

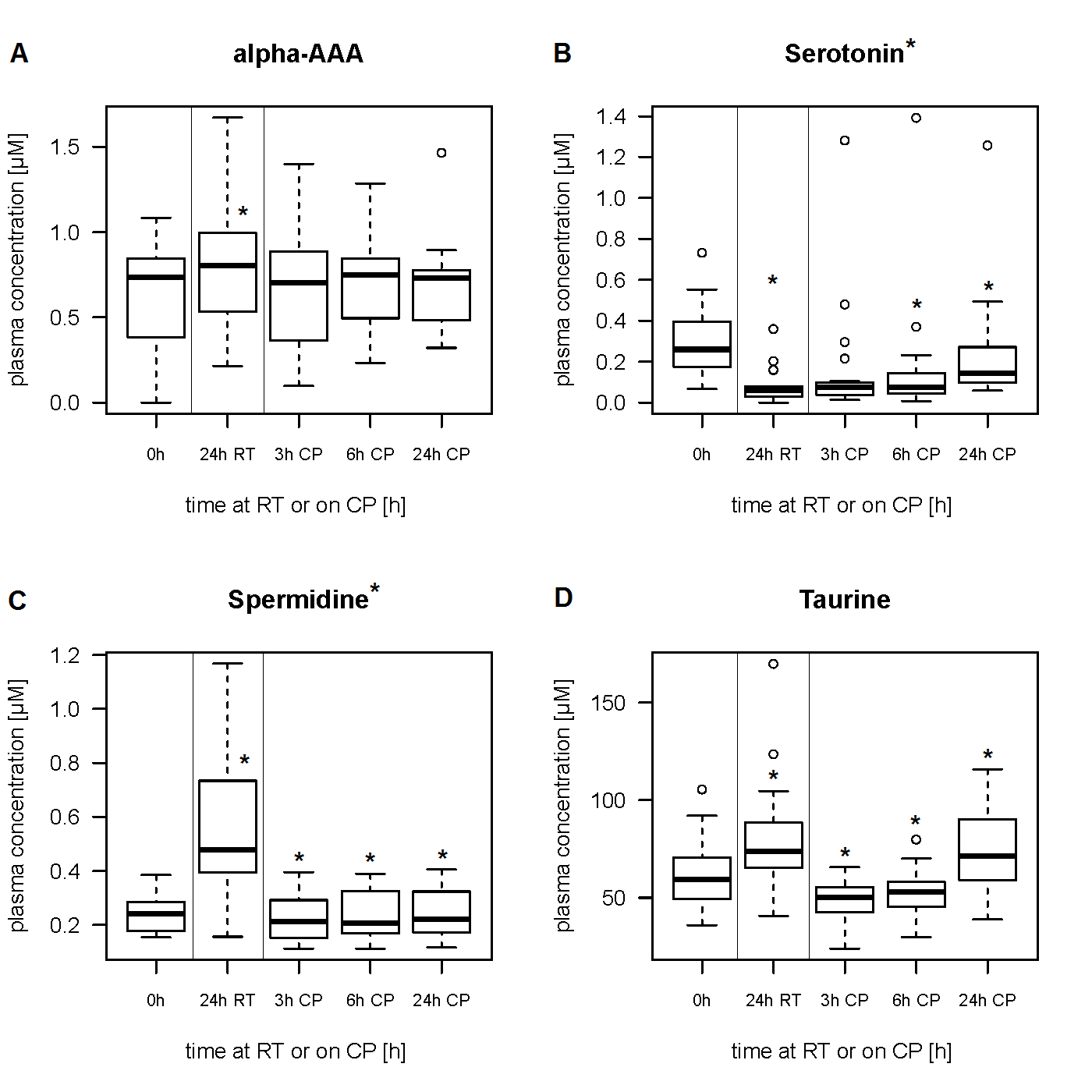

Supplement: Figure S9 — Changes in metabolite concentration during transportation simulation of plasma samples. (A) alpha-AAA, (B) Serotonin*, (C) Spermidine* and (D) Taurine. Stars in boxplots indicate significant difference in concentration compared to baseline (0 h). (Wilcoxon signed rank, significance level p<0.01). (TIFF) [file pone.0089728.s009.tiff]

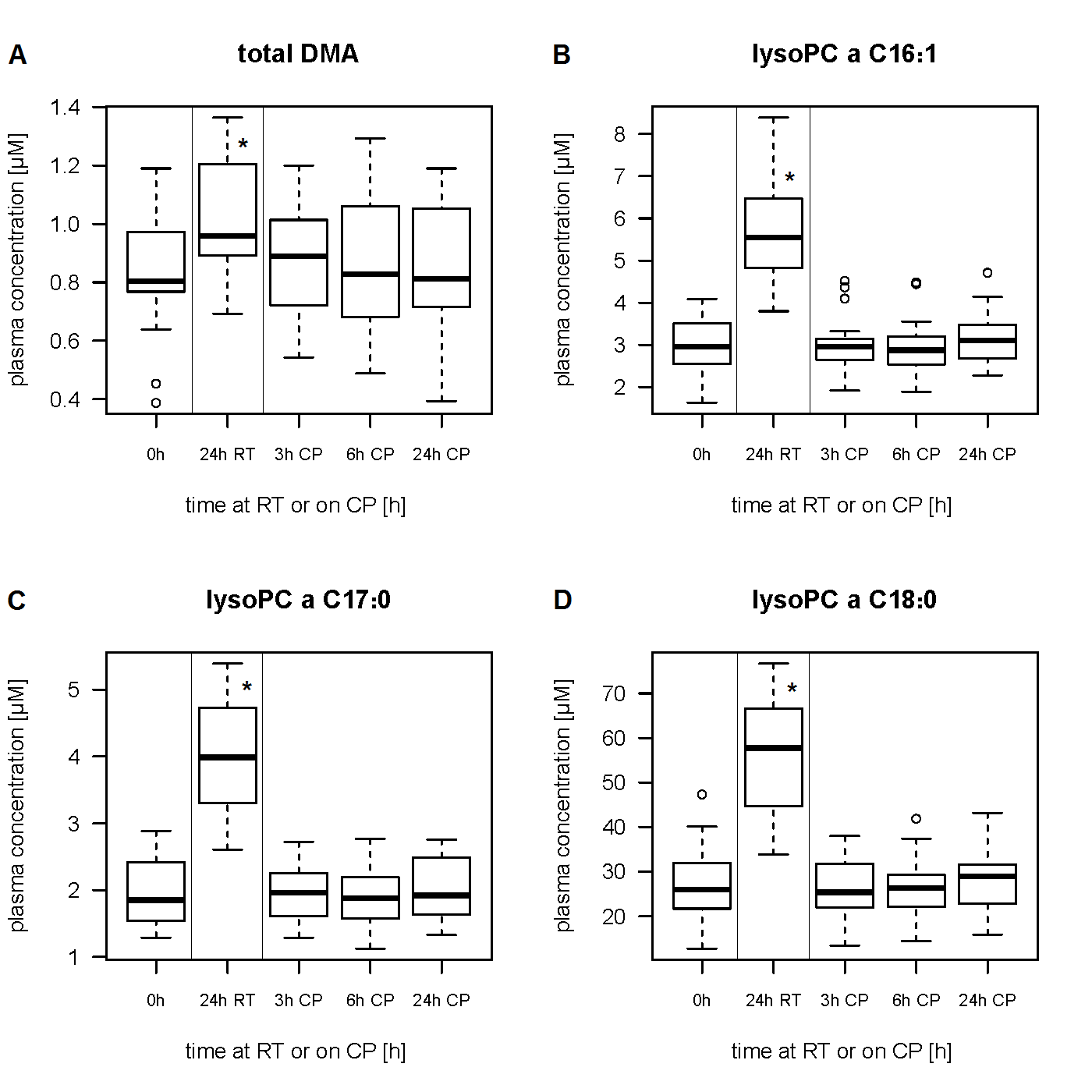

Supplement: Figure S10 — Changes in metabolite concentration during transportation simulation of plasma samples. (A) total DMA, (B) lysoPC a C16∶1, (C) lysoPC a C17∶0 and (D) lysoPC a C18∶0. Stars in boxplots indicate significant difference in concentration compared to baseline (0 h). (Wilcoxon signed rank, significance level p<0.01). (TIFF) [file pone.0089728.s010.tiff]

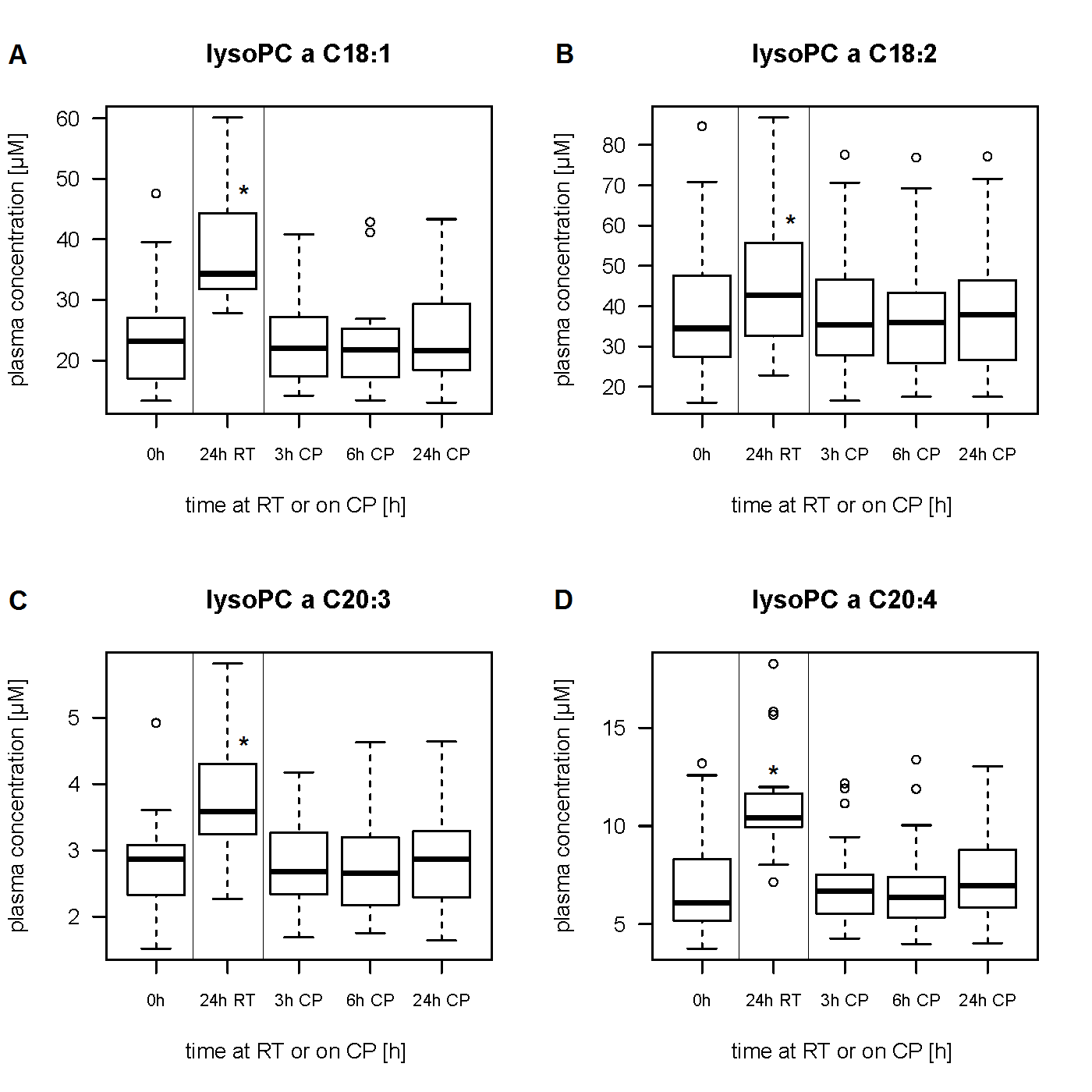

Supplement: Figure S11 — Changes in metabolite concentration during transportation simulation of plasma samples. (A) lysoPC a C18∶1, (B) lysoPC a C18∶2, (C) lysoPC a C20∶3 and (D) lysoPC a C20∶4. Stars in boxplots indicate significant difference in concentration compared to baseline (0 h). (Wilcoxon signed rank, significance level p<0.01). (TIFF) [file pone.0089728.s011.tiff]

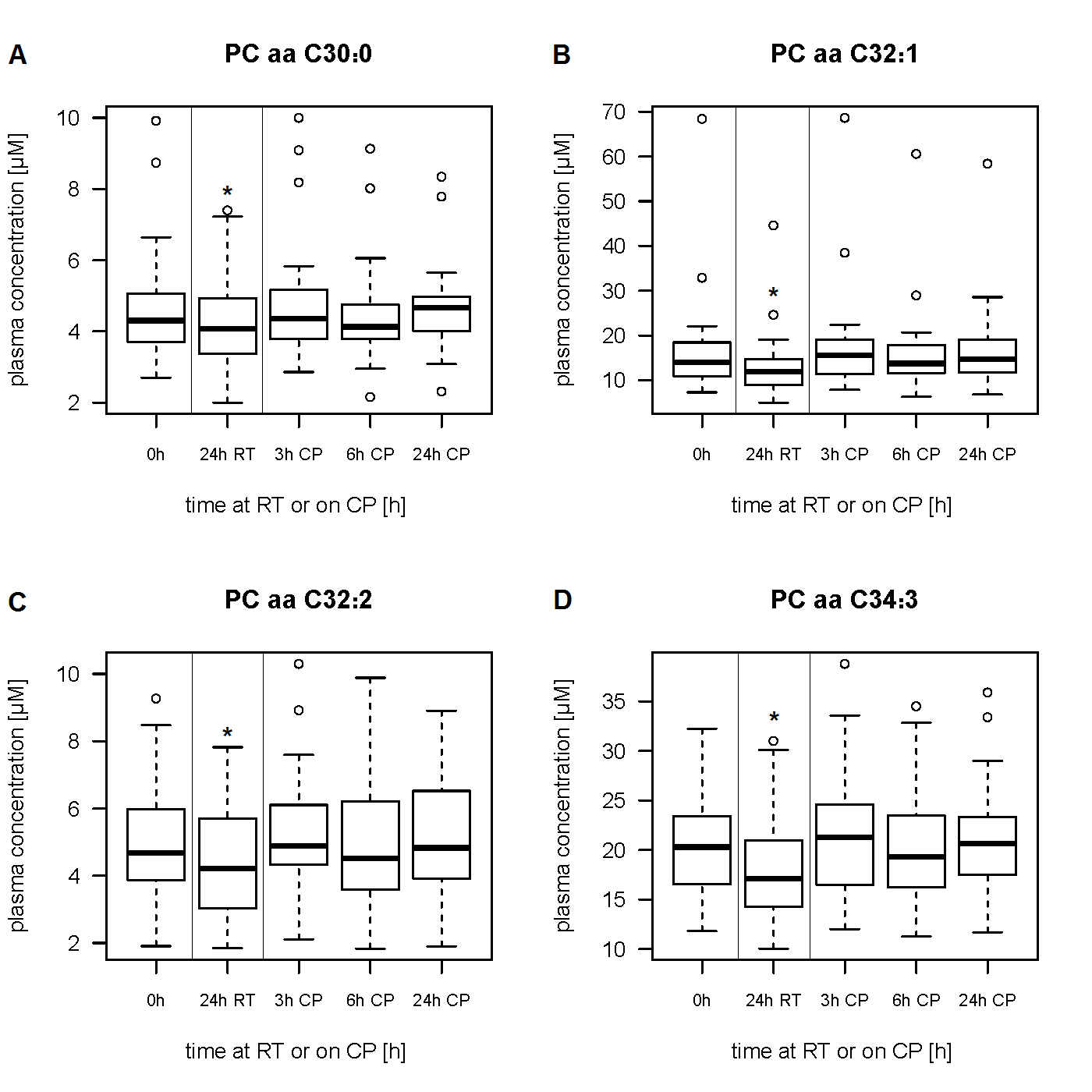

Supplement: Figure S12 — Changes in metabolite concentration during transportation simulation of plasma samples. (A) PC aa C30∶0, (B) PC aa C32∶1, (C) PC aa C32∶2 and (D) PC aa C34∶3. Stars in boxplots indicate significant difference in concentration compared to baseline (0 h). (Wilcoxon signed rank, significance level p<0.01). (TIFF) [file pone.0089728.s012.tiff]

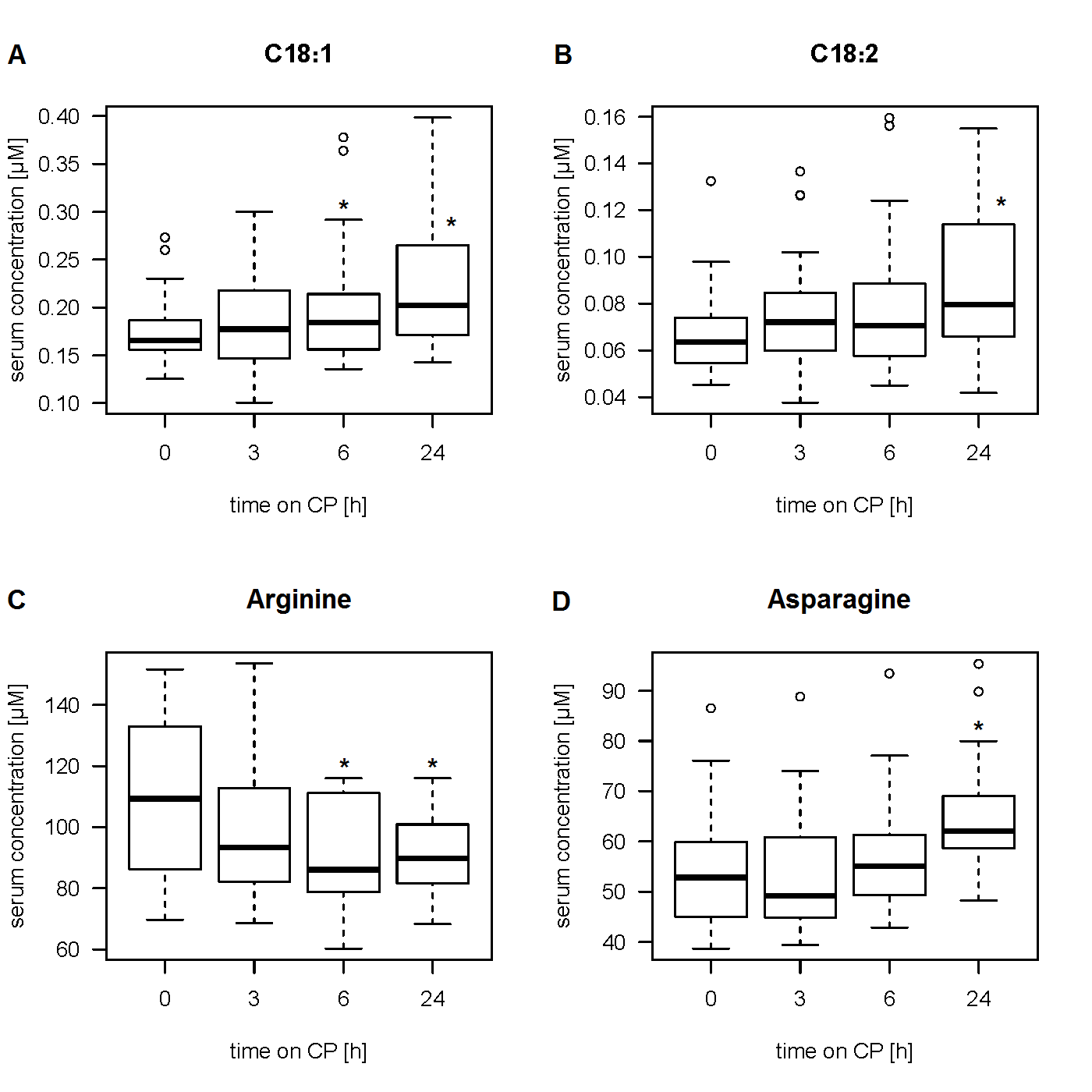

Supplement: Figure S13 — Changes in metabolite concentration during transportation simulation of serum samples. (A) C18∶1, (B) C18∶2, (C) Arginine and (D) Asparagine. Stars indicate significant difference in concentration compared to baseline (0 h). (Wilcoxon signed rank, significance level: p<0.01). (TIFF) [file pone.0089728.s013.tiff]

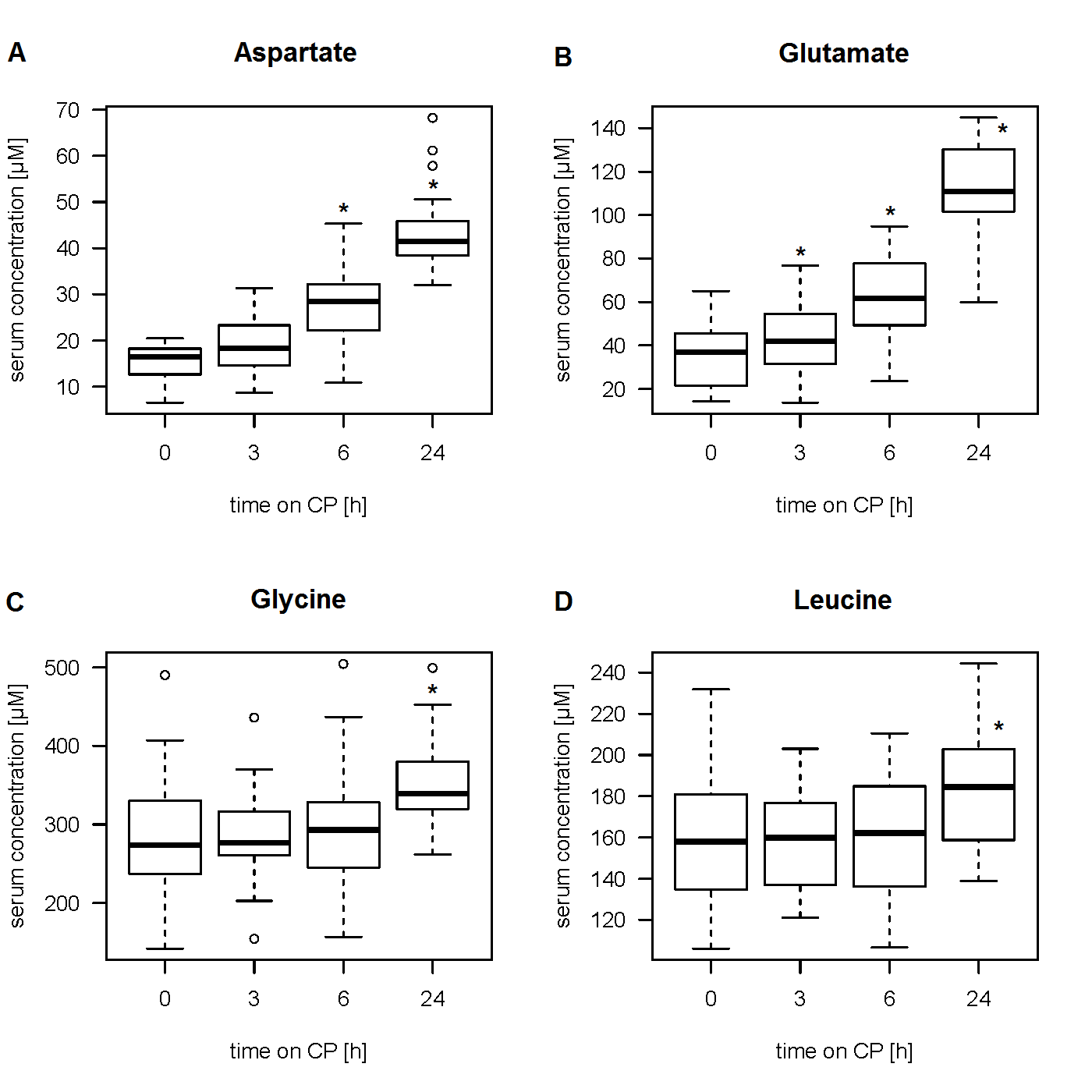

Supplement: Figure S14 — Changes in metabolite concentration during transportation simulation of serum samples. (A) Aspartate, (B) Glutamate, (C) Glycine and (D) Leucine. Stars indicate significant difference in concentration compared to baseline (0 h). (Wilcoxon signed rank, significance level: p<0.01). (TIFF) [file pone.0089728.s014.tiff]

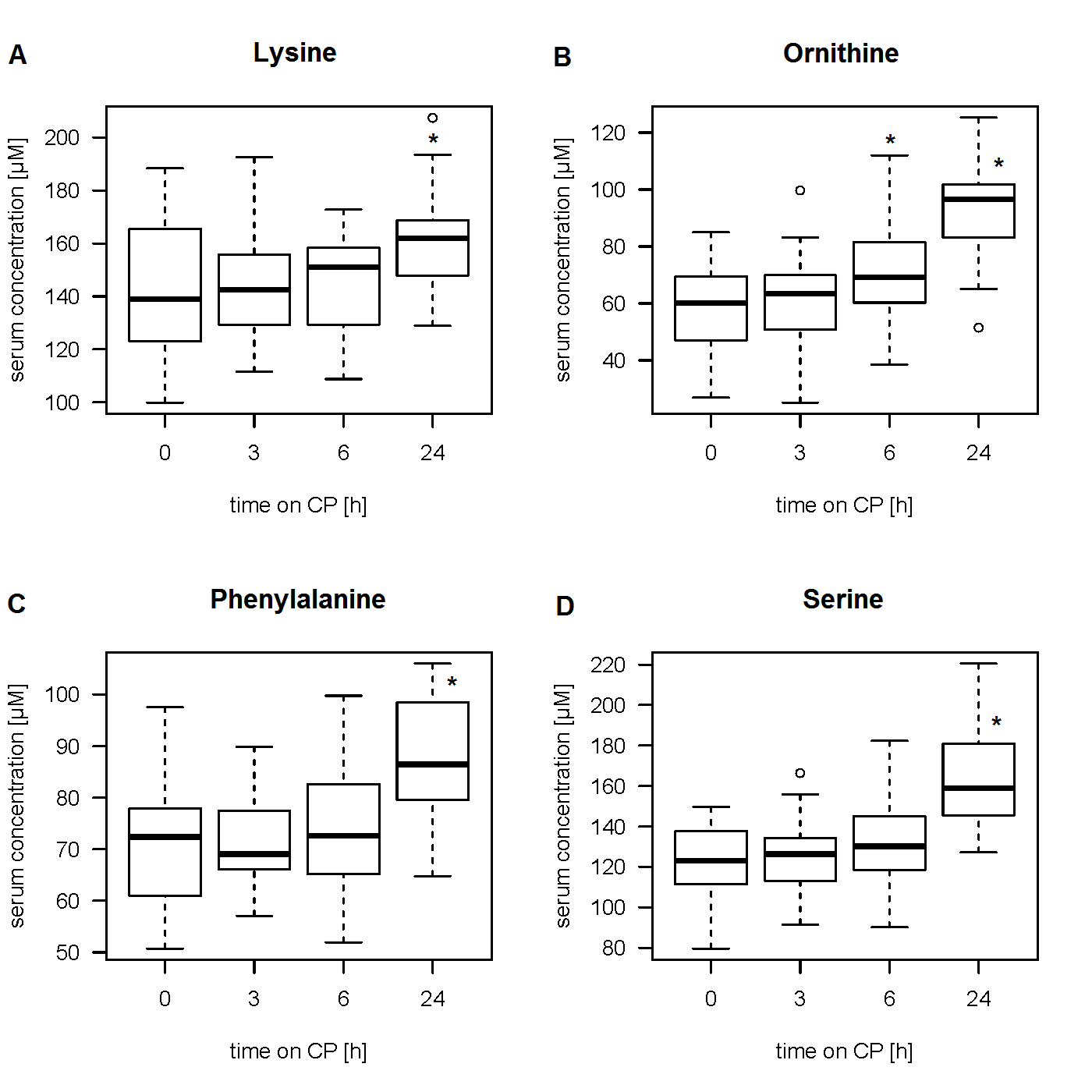

Supplement: Figure S15 — Changes in metabolite concentration during transportation simulation of serum samples. (A) Lysine, (B) Ornithine, (C) Phenylalanine and (D) Serine. Stars indicate significant difference in concentration compared to baseline (0 h). (Wilcoxon signed rank, significance level: p<0.01). (TIFF) [file pone.0089728.s015.tiff]

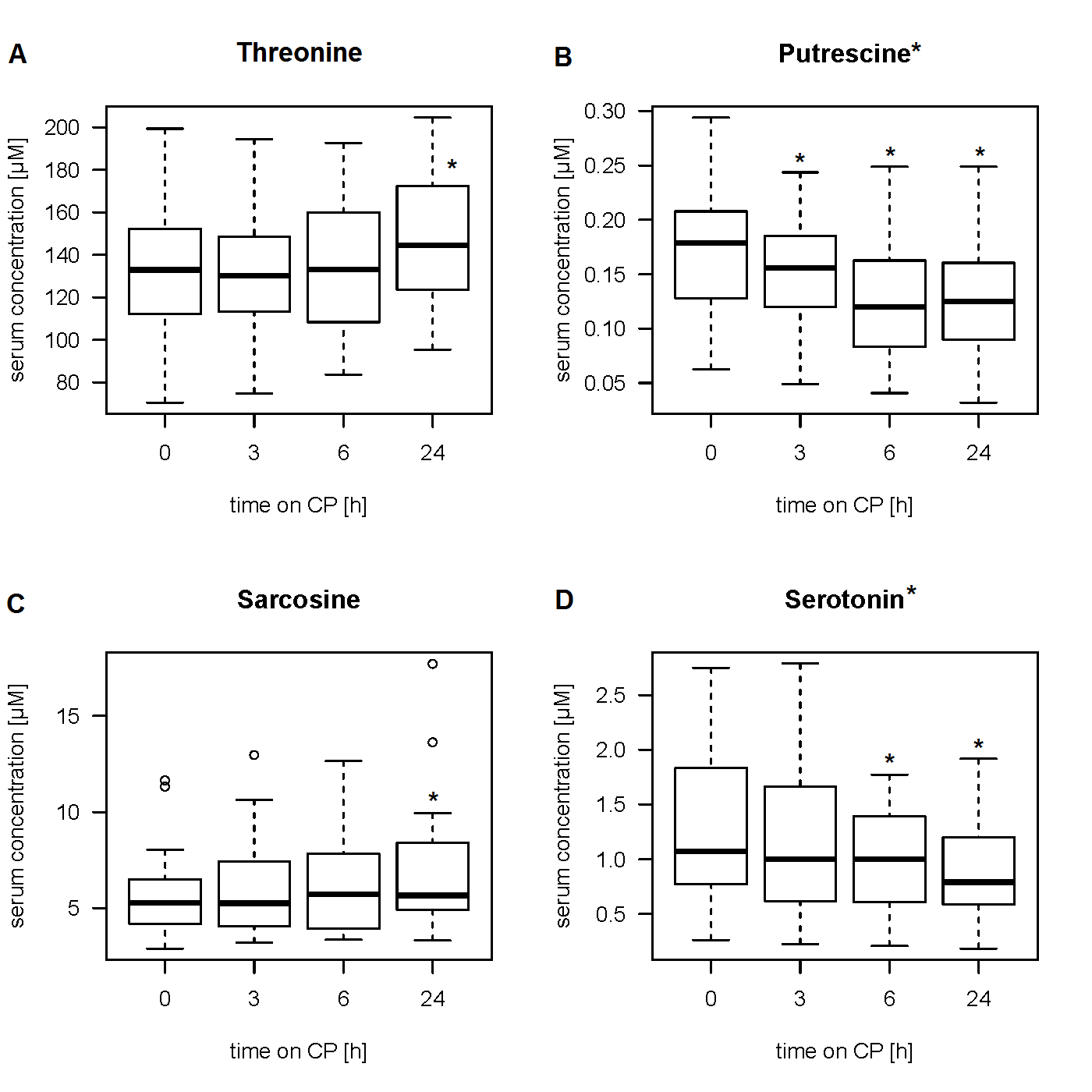

Supplement: Figure S16 — Changes in metabolite concentration during transportation simulation of serum samples. (A) Threonine, (B) Putrescine*, (C) Sarcosine and (D) Serotonin*. Stars indicate significant difference in concentration compared to baseline (0 h). (Wilcoxon signed rank, significance level: p<0.01). (TIFF) [file pone.0089728.s016.tiff]

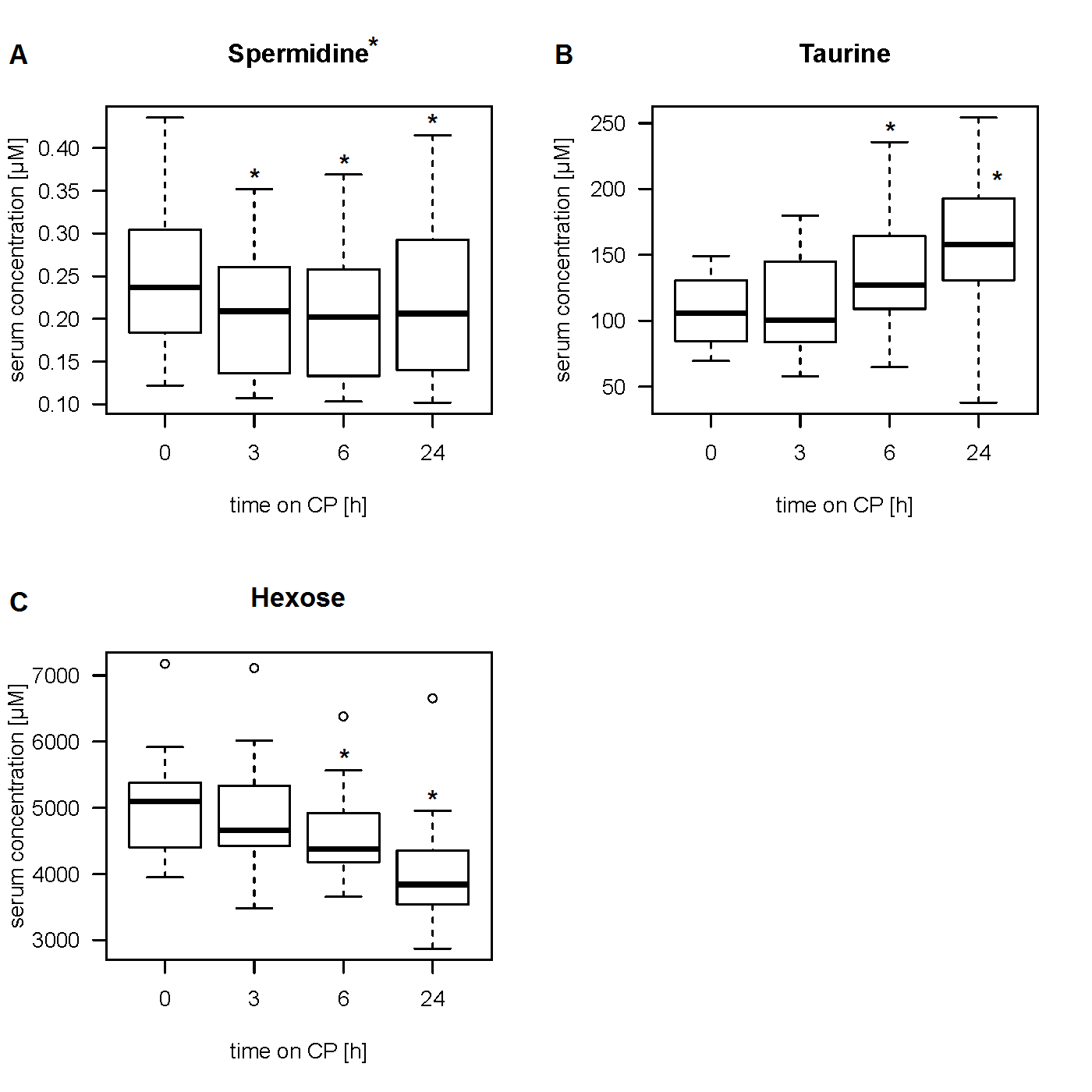

Supplement: Figure S17 — Changes in metabolite concentration during transportation simulation of serum samples. (A) Spermidine*, (B) Taurine and (C) Hexose. Stars indicate significant difference in concentration compared to baseline (0 h). (Wilcoxon signed rank, significance level: p<0.01). (TIFF) [file pone.0089728.s017.tiff]
